# Dengue Fever-Induced Hypokalemic Paralysis in a Pregnant Patient: An Uncommon Presentation of a Common Disease

**DOI:** 10.7759/cureus.40174

**Published:** 2023-06-09

**Authors:** Abu Baker Khan, Muhammad Asfandiyar Ali, Saeed Ur Rehman, Umar Siddiqe, Saad Ahmad

**Affiliations:** 1 Internal Medicine, Ayub Teaching Hospital, Abbottabad, PAK; 2 Internal Medicine, Khyber Teaching Hospital, Peshawar, PAK; 3 Orthopedic Surgery, Taj Medical Center, Nowshera, PAK

**Keywords:** dengue, hypokalemia, acute hypokalemic paralysis, other causes of hypokalemia, recovery of function, potassium replenshment, dengue fever (df)

## Abstract

Dengue fever is a globally prevalent, viral disease transmitted by *Aedes* mosquitoes, which is becoming increasingly common and can cause a range of symptoms, including fever, flu-like symptoms, and circulatory failure. Although it is classified as a non-neurotropic virus, research has suggested that dengue fever can also affect the nervous system and lead to conditions such as myositis, Guillain-Barré syndrome, or hypokalemic paralysis. We describe a case study of a young pregnant female with dengue-associated hypokalemic paralysis, who made a full recovery within 48 hours of receiving potassium supplementation. The case underscores the importance of recognizing and treating neurological complications of dengue fever promptly, particularly in areas where the disease is prevalent.

## Introduction

Dengue fever is a viral disease that is spread by *Aedes* mosquitoes, primarily in tropical and subtropical regions worldwide. It is becoming increasingly common, with 50 to 100 million infections recorded annually by the World Health Organization (WHO). In the year 2021, Pakistan has reported 48,906 cases and 183 deaths due to dengue fever [1]. Dengue fever can affect individuals of any age and can be life-threatening if it develops into severe dengue. Symptoms may include fever, flu-like symptoms, eye pain, dengue hemorrhagic fever, circulatory failure-induced shock, a decrease in platelet count (thrombocytopenia), and low concentration of red blood cells (anemia) [2]. Although classified as a non-neurotropic virus, various studies suggest that dengue may affect the nervous system. Acute neuromuscular weakness is thought to be due to myositis, Guillain-Barré syndrome, or hypokalemic paralysis during dengue. While numerous authors have reported hypokalemic paralysis associated with dengue, most studies are based on case reports [3-6]. We present an intriguing case of hypokalemic paralysis associated with dengue in a young, pregnant female who fully recovered within 48 hours of potassium supplementation.

## Case presentation

A 30-year-old 26-week pregnant female patient presented to the emergency department with complaints of sudden onset of bilateral lower limb weakness, with the inability to walk for about eight hours prior to presentation. This was associated with high-grade fever (103°F), headache, nausea, vomiting, generalized body aches, and joint and muscle pains for three days. On examination, the patient was conscious, and oriented in time, place, and person. The patient's blood pressure was 100/65 mmHg, pulse rate was 108/minute, and oxygen saturation was 96% on room air. The patient was pale, mildly dehydrated, and anxious. On neurological examination, the patient was conscious, and oriented in time, place, and person. There was a decreased power of 3/5 in both lower limbs and a decreased tone. Deep tendon reflexes were absent in both lower limbs. Planter reflexes were normal. There were no signs of a sensory, cranial nerve, or autonomic dysfunction. While on upper limb examination, tone and reflexes were normal with a power of 5/5. The rest of the physical exam was without any relevant findings.

**Figure 1:**
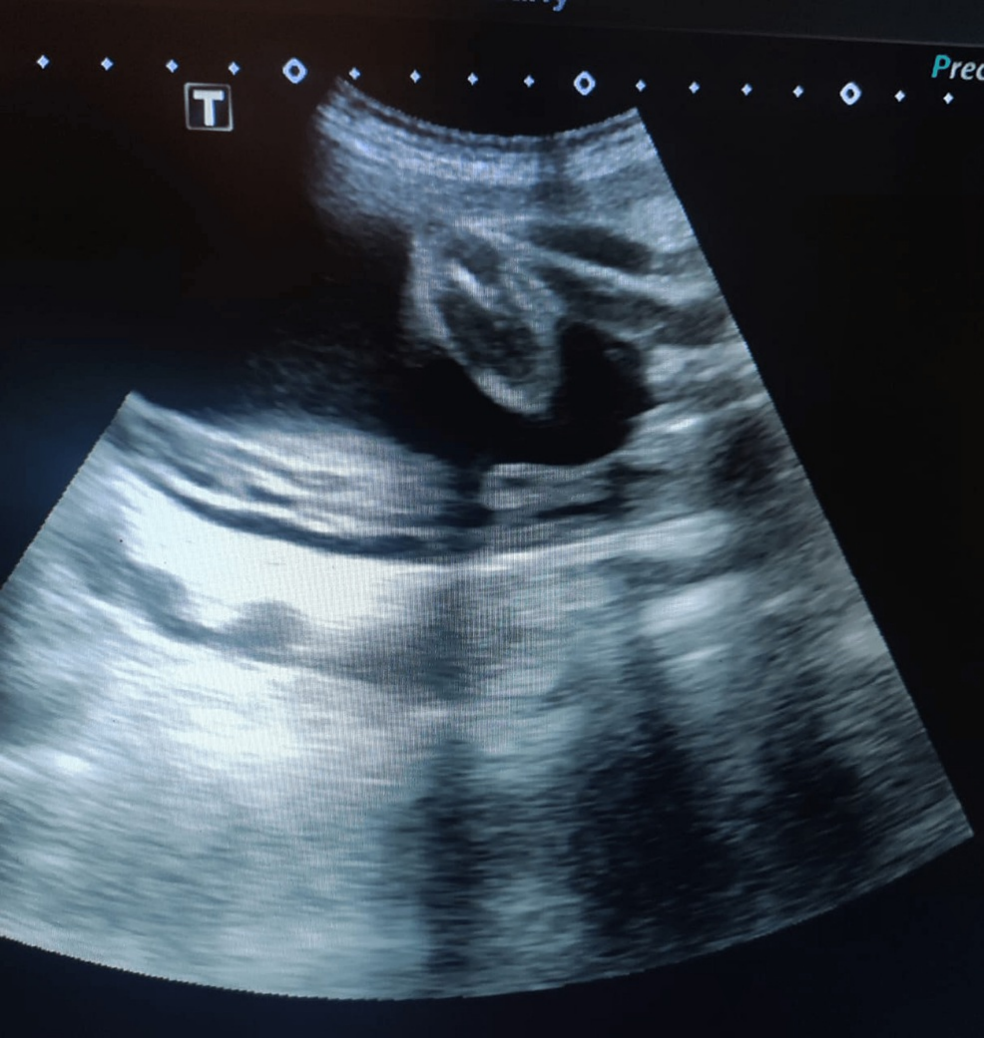
Normal fetal ultrasound at 26 weeks of gestation

Laboratory investigations revealed a white blood cell count of 2700/μL, and platelet count was decreased (88,000/µl). Serum electrolytes showed decreased potassium of 2.5 mmol/L with normal sodium and chloride levels. The rest of the lab findings are mentioned in Table 1. The patient's thyroid profile and random blood glucose level were normal. The patient's dengue NS1 antigen was positive by the immunochromatographic method and the malarial parasite was negative on the rapid immunochromatographic assay. Typhus and leptospirosis were excluded by negative serology. The patient's urinalysis was unremarkable. The patient's nerve conduction study showed normal condition velocity and amplitude of nerve action potential. Based on the patient's history, clinical examination, and laboratory investigations, a diagnosis of dengue fever-associated hypokalemic paralysis was made. The patient was treated with potassium chloride at a rate of 10 mEq/hour in normal saline infusion, which resulted in clinical improvement of lower limb weakness. The patient was also started on oral potassium chloride tablets. On a subsequent day, the patient's electrolytes were normal. Fetal well-being was assessed using ultrasonography (Figure 1), which was normal. The patient was monitored for any electrolyte imbalance and any bleeding for 24 hours and was discharged home in stable condition with no muscle weakness and with normal electrolytes and renal function test.

**Table 1:** Laboratory investigations

	1st day of admission	2nd day of admission	Normal value
White blood cells	2700/µL	3200/µL	4-11/µL
Hemoglobin	10.2 g/dl	10.2 g/dl	11.5-17.5 g/dl
Platelets	88,000/µL	92,000/µL	150-450/µL
Serum sodium	139 mmol/L	143 mmol/L	135-150 mmol/L
Serum potassium	2.57 mmol/L	3.6 mmol/L	3.5-5.1 mmol/L
Blood urea	8.6 mg/dl	14 mg/dl	10-50 mg/dl
Serum creatinine	0.56 mg/dl	0.6 mg/dl	0.42-1.06 mg/dl
Alanine transaminase	162 IU/L	160 IU/L	10-50 IU/L
Creatinine kinase	434 IU/L	370 IU/L	26-140 IU/L

## Discussion

Neurological symptoms associated with dengue fever include encephalitis, encephalopathy, aseptic meningitis, mononeuropathies, polyneuropathies, Guillain-Barré syndrome, myelitis, intracranial hemorrhage, and thrombosis. The development of these symptoms can be attributed to multiple factors, including the neurotropic effect of the dengue virus, the systemic effect of dengue infection, and injury caused by the immune system [7]. Hypokalemia resulting from dengue infection can lead to acute pure motor quadriparesis, but proper potassium correction can result in full recovery.

Our case report involves a pregnant female patient with bilateral lower limb weakness diagnosed with dengue-induced hypokalemic paralysis. When diagnosing hypokalemic paralysis, it is essential to consider Guillain-Barré syndrome as the most important differential diagnosis. Patients with dengue-associated Guillain-Barré syndrome may require intravenous immunoglobulins treatment, whereas those with dengue-associated hypokalemic paralysis typically show improvement soon after potassium is administered. Both conditions present with areflexic quadriparesis, although some asymmetry in reflexes may be observed along with preserved sensations and a flexor plantar response [8]. Normal nerve conduction studies, electromyography, and mildly raised serum creatine phosphokinase excluded Guillain-Barré syndrome and myositis in our patient, which can present similarly.

**Figure 2:**
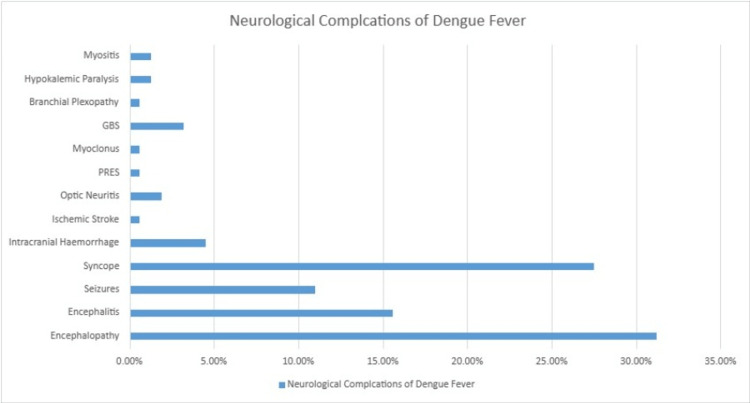
Incidence of neurological complication of dengue fever PRES: posterior reversible encephalopathy syndrome; GBS: Guillain-Barré syndrome. Graph data [10].

In a retrospective analysis of 29 hypokalemic paralysis patients, Garg et al. found that four patients had a history of fever and myalgia and were diagnosed with dengue fever, and they fully recovered with potassium supplementation [9]. In another cross-sectional study conducted in a tertiary care hospital in western India, among 5821 patients diagnosed with dengue, 154 (2.64%) had neurological manifestations, with the most common being encephalopathy, encephalitis, and syncope. Hypokalemic paralysis developed in 1.3% of the patients. This study is the largest reported study of neurological complications due to dengue (Figure 2) [10].

Our case report also highlights the effect of dengue fever on fetal well-being. The potential relationship between dengue fever and fetal congenital malformation is not well established. Sharma et al. reported an increased incidence of neural tube defects (NTDs) in newborns born during the dengue epidemic in a northern district of India, suggesting that the cluster of NTDs is likely due to dengue virus infection [11]. Our patient was 26 weeks pregnant at the time of presentation with good fetal movements. Fetal well-being was assessed using ultrasonography and cardiotocography, which were normal.

Hypokalemic paralysis is a rare complication of dengue fever and other infectious diseases, such as leptospirosis and chikungunya [12]. The exact mechanism of hypokalemia in dengue fever remains unclear, but Jha and Ansari proposed two possible mechanisms for this phenomenon: redistribution of potassium in cells or transient renal tubular abnormality leading to increased urinary potassium excretion. This instance emphasizes the link between hypokalemic paralysis and dengue fever [3]. Medical practitioners need to be familiar with this relationship, particularly in regions such as Pakistan, where atypical signs of dengue fever are increasingly prevalent. This awareness can aid in the timely diagnosis and management of the illness.

## Conclusions

Indeed, this case report highlights the need for healthcare providers to be vigilant for potential neurological complications in patients with dengue fever, especially in regions where dengue is endemic. Additionally, this case emphasizes the importance of prompt diagnosis and treatment of hypokalemic paralysis in patients with dengue fever, as potassium supplementation can lead to a rapid and complete recovery. Finally, this case report underscores the importance of monitoring fetal well-being in pregnant patients with dengue fever, as this infection can potentially cause harm to the fetus. Overall, this case report provides valuable insights into the potential complications and management of dengue fever, particularly in pregnant patients.
